# Sanxan–Whey Protein Isolate Composite-Fabricated Pickering HIPEs: Synergistic Stabilization, Environmental Tolerance and Enhanced Curcumin Bioaccessibility

**DOI:** 10.3390/foods15173121

**Published:** 2026-09-02

**Authors:** Jiangyue Pi, Haiqing Wu, Yang Zhang, Wenzhe Ren, Yanling Xu, Xiaoyan Li, Haidong Huang

**Affiliations:** 1 Tianjin Agricultural University, Tianjin 300392, China; pijiangyue@163.com (J.P.); haiqing_w@yeah.net (H.W.); yangzhang2014@163.com (Y.Z.); 18202519806@163.com (W.R.); xuyanlingtjau@163.com (Y.X.); 2 State Key Laboratory of Non-Food Biomass Energy Technology, Nanning 530007, China

**Keywords:** Sanxan, high internal phase emulsions, whey protein isolate, curcumin

## Abstract

Traditional high internal phase emulsions (HIPEs) require high protein dosage and exhibit poor thermal-saline tolerance, limiting their application as clean-label food carriers for lipophilic nutrients. Sanxan (SAN) possesses unique gelling and emulsifying properties, while its application in HIPE systems remains largely unexplored. This study fabricated curcumin (Cur)-loaded HIPEs using SAN and whey protein isolate (WPI) at lower concentrations. A stable gel network formed at 0.8% SAN and 3% WPI. The oil-binding capacity reached 97.39%, remaining 96.74% and 97.03% after 0.1 M NaCl and 90 °C heating for 30 min. Cur encapsulation efficiency attained 83.23% at 3.5 mg/mL. FTIR verified non-covalent interactions, including hydrogen bonds, hydrophobic and electrostatic forces among SAN, WPI, and Cur. The SAN-WPI (SW)-HIPEs improved the antioxidant activity of Cur; compared with the WPI-HIPEs, ABTS and DPPH radical scavenging capacities increased by approximately 36.94% and 66.22%. In vitro digestion showed merely 1.06% Cur release in gastric fluid; the final intestinal release and bioavailability were 94.77% and 80.46%. This work clarifies the synergistic stabilization mechanism of SAN-WPI binary HIPEs and provides a low-protein formulation strategy to encapsulate and orally deliver lipophilic bioactives for functional food production.

## 1. Introduction

Polysaccharides rank among the most abundant and multifunctional natural biomaterials. They are widely adopted in food, pharmaceutical, and biomedical fields. Their broad application originates from outstanding biocompatibility, tunable gelling capacity, and diverse molecular architectures [1]. Among all polysaccharide subgroups, microbial exopolysaccharides have drawn growing research interest. They possess stable production quality and customizable physicochemical traits, which are hard to replicate from plant-derived raw materials [2]. Sanxan (SAN) is an anionic exopolysaccharide belonging to the sphingan biopolymer family and has been approved as a food additive in China since 2020. Distinct from conventional plant hydrocolloids such as pectin, starch, and carrageenan, SAN integrates four advantageous traits simultaneously. It exhibits high intrinsic viscosity, prominent shear-thinning flow behavior, thermally irreversible gelation, and inherent interfacial activity [3]. These unique physicochemical features support its existing application scenarios, including drug-loaded microcapsules [4], green adsorbents [5], and controlled-release hydrogel fertilizers [6]. Such findings also validate its great potential as a multifunctional food-compatible biomacromolecule. Even so, few studies explore the possibility of SAN serving as the primary structural matrix in emulsion systems, especially when combined with protein molecules.

Given the outstanding gelling and interfacial performance of SAN, this microbial polysaccharide holds great promise for fabricating food-grade delivery carriers and fat substitutes [7,8]. Among various colloidal delivery platforms, hydrogels [9] and nanoparticles [10] have been widely explored for lipophilic bioactive encapsulation, yet each system faces challenges such as low loading capacity and high production costs for food applications. High internal phase emulsions (HIPEs, *φ* ≥ 0.74), in contrast, offer advantages in high loading capacity and desirable rheological properties, making them particularly suitable for low-calorie food applications [11]. However, the translation of HIPEs into practical food applications is severely hindered by several interconnected challenges. Most stable HIPEs require protein concentrations above 5% (*w*/*v*), which inevitably increases production costs and the caloric load of final products. Single protein systems also tend to aggregate near isoelectric points or under high ionic strength, which triggers rapid demulsification [12,13]. In addition, polysaccharide particles alone exhibit insufficient interfacial activity and often require synthetic surfactants as emulsification auxiliaries [14]. Such additions are discouraged in clean-label product development. Collectively, these limitations significantly restrict the large-scale application of protein–polysaccharide HIPEs in the current food industry landscape. To overcome these limitations, polysaccharide–protein composite systems have been widely explored as HIPE stabilizers [15,16]. Nevertheless, most reported composites rely on conventional polysaccharides such as chitosan, pectin, or carrageenan. These systems still require high protein dosages and show limited resistance to combined thermal and saline stresses [17,18,19]. In view of the distinctive properties described above, Sanxan offers a promising alternative for constructing HIPEs with reduced protein dependence. Whey protein isolate (WPI) is selected as the protein counterpart due to its excellent nutritional quality, emulsifying activity, and widespread use in food formulations [20]. Previous studies have confirmed that SAN can form composite particles with proteins through non-covalent interactions, improving the stability of Pickering emulsions [21]. Little research has explored SAN-WPI composite HIPEs, and it remains unconfirmed whether such systems can achieve low-WPI formulation, strong resistance to thermal-saline stress, and controlled release of lipid-soluble functional ingredients.

In this study, curcumin (Cur) was employed as a model lipophilic nutrient to evaluate the delivery performance of SAN-WPI HIPEs. Cur is well known for its antioxidant and anti-inflammatory properties, yet its poor water solubility, rapid photodegradation, thermal lability, and low oral bioavailability severely limit its food applications [22,23,24]. This study systematically explores how WPI dosage modulates the structure and stability of HIPEs, evaluates the thermal and saline tolerance of the emulsions, and characterizes the protection and release performance of Cur during simulated gastrointestinal digestion. The outcomes offer mechanistic understanding of SAN-protein synergistic stabilization in HIPEs and support the development of low-protein formulations for delivering lipophilic bioactive compounds in functional foods.

## 2. Materials and Methods

### 2.1. Materials

Food-grade sanxan was purchased from Hebei Fengchuan Biotechnology Co., Ltd. (Hebei, China; Molecular weight: 4.08 × 10^5^ Da, and the molecular mass dispersity value was 1.07) [25]. Lipase (20,000 U/g) and curcumin (≥95%) were purchased from Shanghai Meiruier Biochemical Technology Co., Ltd. (Shanghai, China). Soybean oil (refined, non-GMO; brand: Jinlongyu) was purchased from Kerry Grain and Oil (Tianjin) Co., Ltd. (Tianjin, China). Pepsin (porcine gastric mucosa, 1:10,000) was purchased from Shanghai Yuanye Biotechnology Co., Ltd. (Shanghai, China). Whey protein isolate (80%) and Trypsin (porcine pancreas, 1:250) were purchased from Shanghai Maclean’s Biochemical Science and Technology Co., Ltd. (Shanghai, China). Bile salts were purchased from Sigma-Aldrich (St. Louis, MO, USA). All other chemicals used in the experiments were of analytical grade.

### 2.2. Fabrication of SAN-WPI (SW) Complex

Dissolve 0.8 g of SAN in 100 mL of deionized water. After complete dissolution on a magnetic stirrer, add WPI (1 g, 2 g, 3 g, 4 g, and 5 g) and stir overnight to obtain SW mixed solutions (1%, 2%, 3%, 4%, and 5%; *w*/*v*, abbreviated as SW1%, SW2%, SW3%, SW4%, and SW5%). The blank consisted of 0.8 wt% SAN (the polysaccharide control with fixed concentration) and 5 wt% WPI (the highest protein dosage control).

### 2.3. Characterization of SW Complex

#### 2.3.1. Particle Size and Zeta Potential

The average particle size, polydispersity index (PDI), and zeta potential of the complexes were determined using dynamic light scattering [26]. The measurements were performed using a Malvern Nano-ZS90 laser particle size analyzer (Malvern Instruments Ltd., Worcester, UK) at a temperature of 25 °C.

#### 2.3.2. Three-Phase Contact Angle

Compress the dried sample at room temperature into a thin film approximately 1–2 mm thick and 10.0 mm in diameter. Precisely place a 5 µL drop of pure water on the sample surface, measure the three-phase contact angle (θ), and record the droplet shape using a Theta optical contact angle measuring instrument (Biolin, Gothenburg, Sweden).

### 2.4. Preparation of SW-HIPEs and Optical Microscope

After mixing soybean oil and the complex (prepared as described in Section 2.2) in an 8:2 (*v*/*v*) ratio, SW-HIPEs were prepared using a high-shear disperser (T25 digital, IKA, Staufen, Germany) at 16,000 rpm for 1 min. A small amount of the emulsion was observed under an optical microscope (BA210, Motic, Xiamen, China) using a 40× objective lens. Curcumin was pre-dissolved in the oil phase, and SW-HIPEs-Cur was prepared using the same method as described above.

### 2.5. The Oil Binding Capacity (OBC)

The OBC is used to evaluate the oil immobilization ability of HIPE gel networks, reflecting the capacity of the gel matrix to confine the oil phase within the internal network. Specifically, 10 g of each sample was placed in a 50 mL centrifuge tube and centrifuged at 6000 rpm for 10 min at 4 °C. The OBC of HIPEs was calculated by the following Formula (1):

(1) $$\mathrm{OBC}\left(\%\right)=(1-\frac{{W_{i}-W}_{f}}{W_{i}})\times 100\%$$

where W_i_ represented the weight of the sample before centrifugation, and W_f_ represented the weight of oil removed after centrifugation.

### 2.6. Rheological Measurements

The rheological properties of SW-HIPEs were characterized using a dynamic shear rheometer (MCR302, Anton Paar, Ostfildern-Scharnhausen, Germany) at 25 °C. Following the method reported by [27], the apparent shear viscosity and loss modulus of the HIPEs were measured at frequencies ranging from 0.1 to 100 rad/s and a strain of 1%.

### 2.7. Scanning Electron Microscopy (SEM)

Following the method described in [28], the samples were dehydrated in a vacuum freeze-dryer for 6 h, then soaked in n-hexane at room temperature overnight, and subsequently dried in an oven at 50 °C for 4 h. After 200 s of gold sputtering, SEM images were acquired using a scanning electron microscope (Sigma 300, Carl Zeiss AG, Oberkochen, Germany).

### 2.8. SW-HIPEs Stability

#### 2.8.1. Ionic Strength

The SW complex (100 mL) contained different concentrations of salt (0.1 M, 0.2 M, 0.3 M, and 0.4 M NaCl), and they were used to fabricate SW-HIPEs. The OBC of SW-HIPEs was determined according to Section 2.5.

#### 2.8.2. Thermal Treatment

Place the centrifuge tube containing 10 g of SW-HIPEs in a water bath and maintain it at 25, 37, 60, and 90 °C for 30 min each; then place it in an ice-water bath to cool to room temperature. The OBC of SW-HIPEs was determined according to Section 2.5.

#### 2.8.3. Storage Stability

After storing the centrifuge tube containing 10 g SW-HIPEs at 4 °C for 30 days, determine the OBC of SW-HIPEs according to Section 2.5.

### 2.9. Encapsulation Efficiency (EE) of HIPEs-Cur

We then centrifuged at 10,000 g for 30 min. The supernatant was collected, and a fluorescence spectrophotometer (FL970, Shanghai Techcomp Scientific Instrument Co., Ltd., Shanghai, China) was employed to determine the fluorescence intensity at an excitation wavelength (Ex) of 442 nm and an emission wavelength (Em) of 475 nm [29]. Cur content was calculated using the Cur standard curve, and the EE of Cur was calculated using the following Formula (2):

Herein, C_f_ is the amount of free Cur in the ethanol solution, and C_t_ is the theoretical

(2) $$\mathrm{EE}\left(\%\right)=\frac{{C_{t}-C}_{f}}{C_{t}}\times 100\%$$

Cur concentration in HIPEs.

### 2.10. Fourier Transform Infrared Spectroscopy (FTIR)

Freeze-dried samples of SW-HIPEs were uniformly mixed with dried KBr powder and pressed into tablets; infrared spectra were then obtained using an FTIR spectrometer (Nicole 6700, Thermo Nicole Company, Waltham, MA, USA). With air as the background, FTIR spectra were recorded over a range of 4000–5000 cm^−1^ using 32 scans, with a resolution of 4 cm^−1^.

### 2.11. SW-HIPEs-Cur Stability

#### 2.11.1. Ultraviolet (UV) Light and Thermal Stability

SW-HIPEs-Cur were exposed to UV light (254 nm, 12 W) for 4 h to determine their photostability. Thermal stability was assessed by placing the samples in a 90 °C water bath; the blank consisted of oil and WPI containing an equal amount of Cur. The Cur content was measured every 0.5 h using the following Formula (3):

(3) $$\mathrm{Retention}\;\mathrm{rate}\left(\%\right)=\frac{C_{T}}{C_{i}}\times 100\%$$

where C_T_ represents the concentration of the treated sample, and C_i_ represents the concentration of the initial sample.

#### 2.11.2. Antioxidant Capacities

##### ABTS Free Radical Scavenging Activity

Following the method described by [30] with minor modifications, 20 µL of the sample was mixed with 1 mL of ABTS working solution. After thorough mixing, the mixture was allowed to react in the dark at room temperature for 6 min. Measure the absorbance of samples at different concentrations after reaction with ABTS at a wavelength of 734 nm. Additionally, mix 20 µL of anhydrous ethanol with 1 mL of ABTS solution and measure the absorbance of the blank group. The calculation Formula (4) is as follows:

(4) $$A\mathrm{BTS}\;\mathrm{scavenging}\;\mathrm{rate}\left(\%\right)=1-\frac{A_{s}}{A_{c}}\times 100\%$$

Herein, A_s_ represents the absorbance of the mixture of ABTS and the sample, and A_c_ represents the absorbance of the ABTS–ethanol mixture.

##### DPPH Free Radical Scavenging Activity

Following the method described by [10] with minor modifications, a 0.05 mM DPPH–ethanol solution was prepared and stored in the dark. Freshly prepared samples were mixed with this solution in equal volumes and allowed to react in the dark at room temperature for 0.5 h, and the absorbance of the reaction solution was measured at 517 nm. The calculation Formula (5) is as follows:

(5) $$\mathrm{DPPH}\;\mathrm{scavenging}\;\mathrm{rate}\left(\%\right)=1-\frac{D_{i}}{D_{0}}\times 100\%$$

where D_i_ represents the absorbance of the mixture of DPPH and ethanol with the sample; D_0_ represents the absorbance of the DPPH–ethanol mixture.

### 2.12. Stability and Bioaccessibility of SW-HIPEs-Cur in Vitro Digestion

#### 2.12.1. In Vitro Digestion Model

The simulated solution was prepared according to the methods described in [31], and the release rate of Cur in the simulated gastrointestinal tract was evaluated. A 1g SW-HIPEs-Cur was placed in a vial containing 6 mL of simulated gastric fluid, then shaken horizontally on a shaker in the dark for 2 h at 37 °C and 120 rpm. Samples were collected every 0.5 h for analysis. After 2 h, the gel was removed and placed into a 6 mL vial containing intestinal simulation solution, where it was simulated for 3 h. Samples were collected every 0.5 h to observe the morphological changes and release characteristics of the gel in different simulated environments. Take 1 mL of the corresponding digestive fluid, centrifuge at 10,000 *g* for 5 min, and then add an equal volume of anhydrous ethanol to the supernatant. Determine the Cur content in the simulated fluid using the method described in Section 2.9, and calculate the rate of release of Cur in the digested solution.

#### 2.12.2. Bioaccessibility

After digestion, centrifuge at 10,000 *g* for 30 min to obtain the micellar phase of the sample. Mix 1 mL of the original digestion product or micellar phase with 4 mL of 95% ethanol until homogeneous. Centrifuge again at 10,000 *g* for 30 min, then measure the absorbance of the supernatant. The calculation Formula (6) is as follows:

(6) $$\mathrm{Bioaccessibility}\left(\%\right)=\frac{C_{1}}{C_{0}}\times 100\%$$

where C_1_ represents the content of Cur in the supernatant, and C_0_ represents the content of Cur in the initial HIPEs.

### 2.13. Statistical Analysis

All experiments were performed at least in triplicate. Duncan tests, one-way analysis of variance, and SPSS (26 version, USA) were used to evaluate the results (Significantly: *p* < 0.05).

## 3. Results

### 3.1. Characterization of SW Complex

#### 3.1.1. Particle Size and Zeta Potential

**Figure 1:**
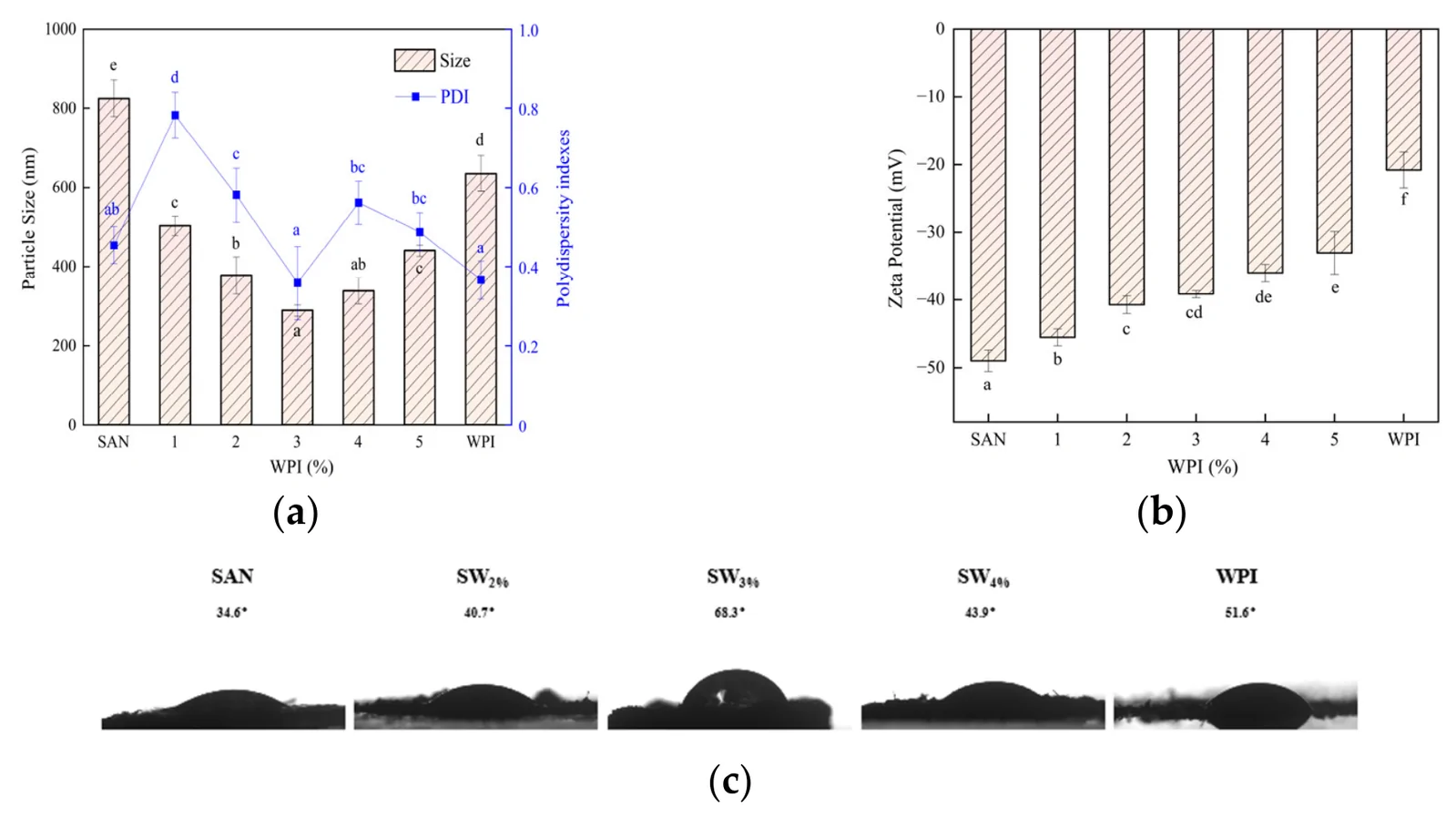
The interaction and structural properties of SW complexes: Mean particle sizes and polydispersity indexes (PDI) (**a**). Zeta potentials (**b**). Three-phase contact angle (θ) (**c**). Free SAN and WPI were used as controls. Different small superscript letters (a–f) indicate the presence of statistically significant differences (*p* < 0.05).

Particle size significantly affects the physical stability of emulsions and the quality of their gels. Figure 1a shows the particle size distribution of solutions formed by SAN with WPI at different concentrations. As illustrated in the Figure, particle size and PDI exhibit distinct trends with increasing WPI concentration. When the WPI concentration is below 2%, insufficient protein fails to fully cover the oil/water interface, leading to easy droplet coalescence, larger particle sizes, and a significant increase in PDI. When the WPI concentration is increased to 2–5%, proteins adsorb at the interface to form a dense, stable Pickering layer, resulting in decreased and stabilized particle size and PDI.

ζ-potential measurements are commonly used to characterize the surface charge of proteins and are widely employed to assess the strength of repulsive interactions between macromolecules in protein-rich colloidal systems. According to [32], a high ζ-potential favors protein dispersion, thereby promoting the formation of a uniform and stable gel system, whereas a low ζ-potential has the opposite effect. As shown in Figure 1b, the ζ-potential exhibits a monotonically decreasing trend. As the WPI concentration increases from 0 to 5%, the absolute value of the ζ-potential continuously decreases, which is consistent with the findings of [33]. Protein molecules adsorbed at the interface endow the droplets with higher charge density, enhancing electrostatic repulsion and thereby improving the stability of the system.

#### 3.1.2. Three-Phase Contact Angle

The interfacial wettability of the complex, determined by the contact angle (θ), is a critical factor influencing the formation and stability of HIPEs. This wettability enhances the adsorption of particles at the oil/water interface and inhibits oil droplet aggregation through steric hindrance effects [34]. When the contact angle of the particles approaches 90°, their interfacial adsorption capacity is maximized, resulting in a more stable Pickering emulsion [35]. The static water contact angle (θ) of the SW complex first increases and then decreases with increasing WPI concentration (Figure 1c). Among all WPI addition groups, SW3% exhibits the largest contact angle and the strongest hydrophobicity, indicating that the formed HIPEs are more stable.

### 3.2. Physicochemical Properties of SW-HIPEs

#### 3.2.1. Optical Microscope

**Figure 2:**
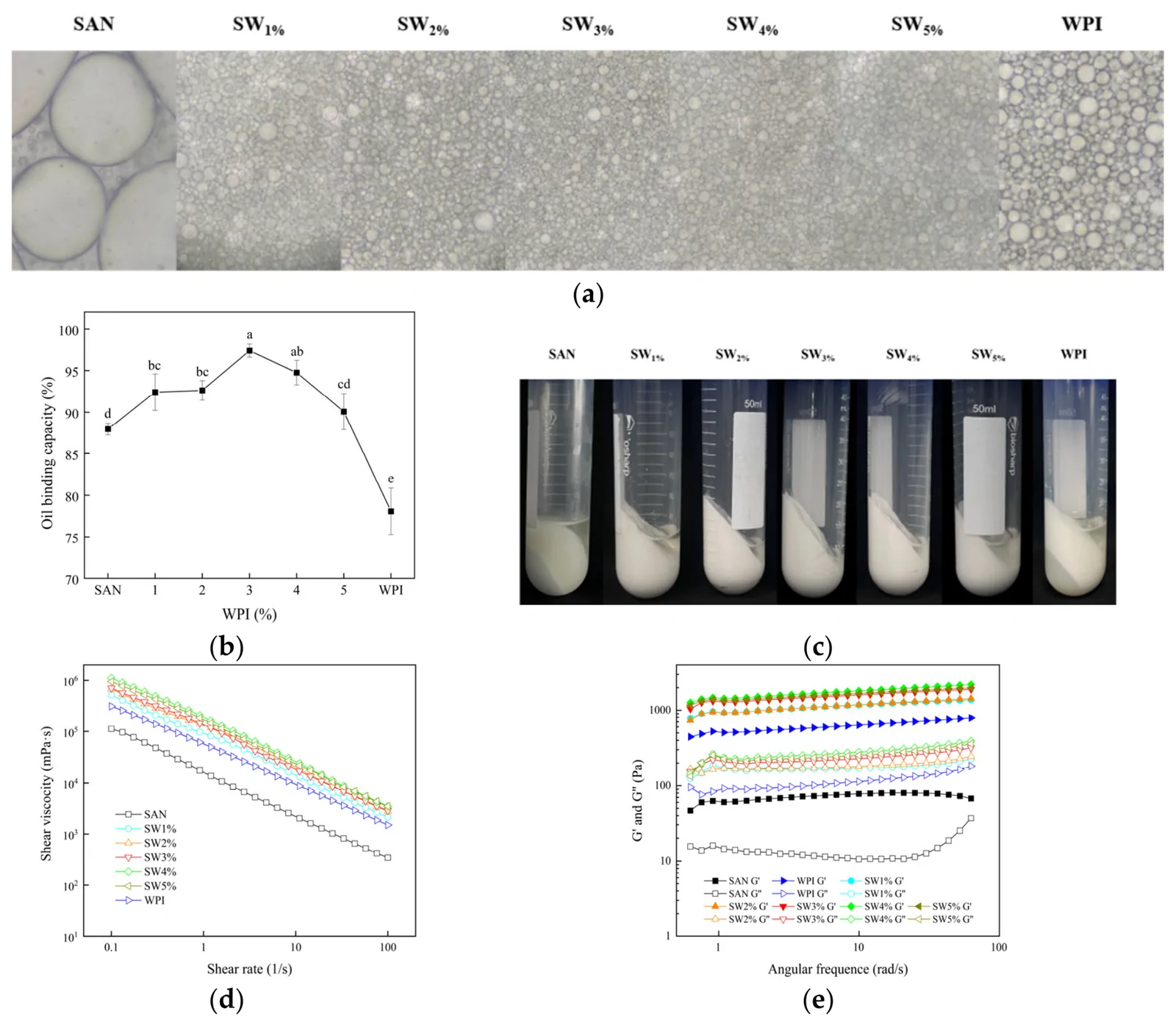
Characterization of SW-HIPEs, which was labeled SW in the manuscript: Optical microscope (**a**). The OBC (**b**). Appearance (**c**). Viscosity (**d**). Frequency scanning (**e**). Different small superscript letters (a–e) indicate the presence of statistically significant differences (*p* < 0.05).

The microscopic morphology of SW-HIPEs observed under an optical microscope is shown in Figure 2a. Compared with the single SAN or WPI system, the composite of the two significantly reduces the particle size of HIPEs. Moreover, different WPI addition amounts are crucial for the formation of HIPEs, affecting their stability and gel network structure. After complexation, all droplets exhibit a rounded morphology. Among the tested groups, the 3% and 4% WPI groups display the smallest and most uniform oil droplets, which is consistent with the particle size data presented earlier. This morphological observation indicates that the optimal WPI concentration facilitates the formation of a denser and more uniform droplet packing.

#### 3.2.2. The OBC

The composite skeleton formed by SAN and WPI endows the network with higher rigidity, enabling proteins to achieve irreversible stability at the oil/water interface in the form of soft particles and thus following the Pickering stabilization mechanism. As shown in Figure 2b,c, the OBC first increases and then decreases with increasing WPI concentration. At a WPI concentration of 1%, the relatively low protein content limits its ability to encapsulate and bind oil droplets, resulting in an oil binding capacity of 92.39%. When the WPI concentration increases to 3% and 4%, the protein forms a dense adsorption layer at the interface, which better encapsulates the oil droplets, raising the oil binding capacity to 97.39% and 94.75%, respectively. Further increasing the protein content does not significantly improve the oil binding rate, and this trend is consistent with the particle size and microscopic examination results.

#### 3.2.3. Rheological Measurements

Dynamic rheological analysis revealed the relationship between viscosity and shear rate for each sample, as shown in Figure 2d. All samples exhibited shear-thinning behavior, characterized by a decrease in viscosity with increasing shear rate, which is a typical rheological feature of HIPE systems [36]. Notably, the viscosity of the SW complex is significantly higher than that of systems containing only SAN or only WPI, and it exhibits a trend of first increasing and then decreasing with increasing WPI concentration, peaking in the 4% WPI group. Figure 2e shows the storage modulus (G′) and loss modulus (G″) as functions of angular frequency for each sample. Within the tested frequency range, G′ remains consistently higher than G″ for most samples, and the loss tangent (tan δ) is less than 0.25, indicating that these samples possess gel-like elastic solid characteristics, with their internal network structures dominated by elastic responses. Considering the oil binding capacity results, the composite system with 3% WPI, despite having a slightly lower modulus than systems with higher protein concentrations, exhibits a more uniform interfacial protein distribution. This system achieves a higher oil binding capacity while using less protein, thereby striking an optimal balance between gel strength and formulation cost. Consequently, it demonstrates the best overall performance, and the SW3% was selected for subsequent experiments.

#### 3.2.4. SEM

**Figure 3:**
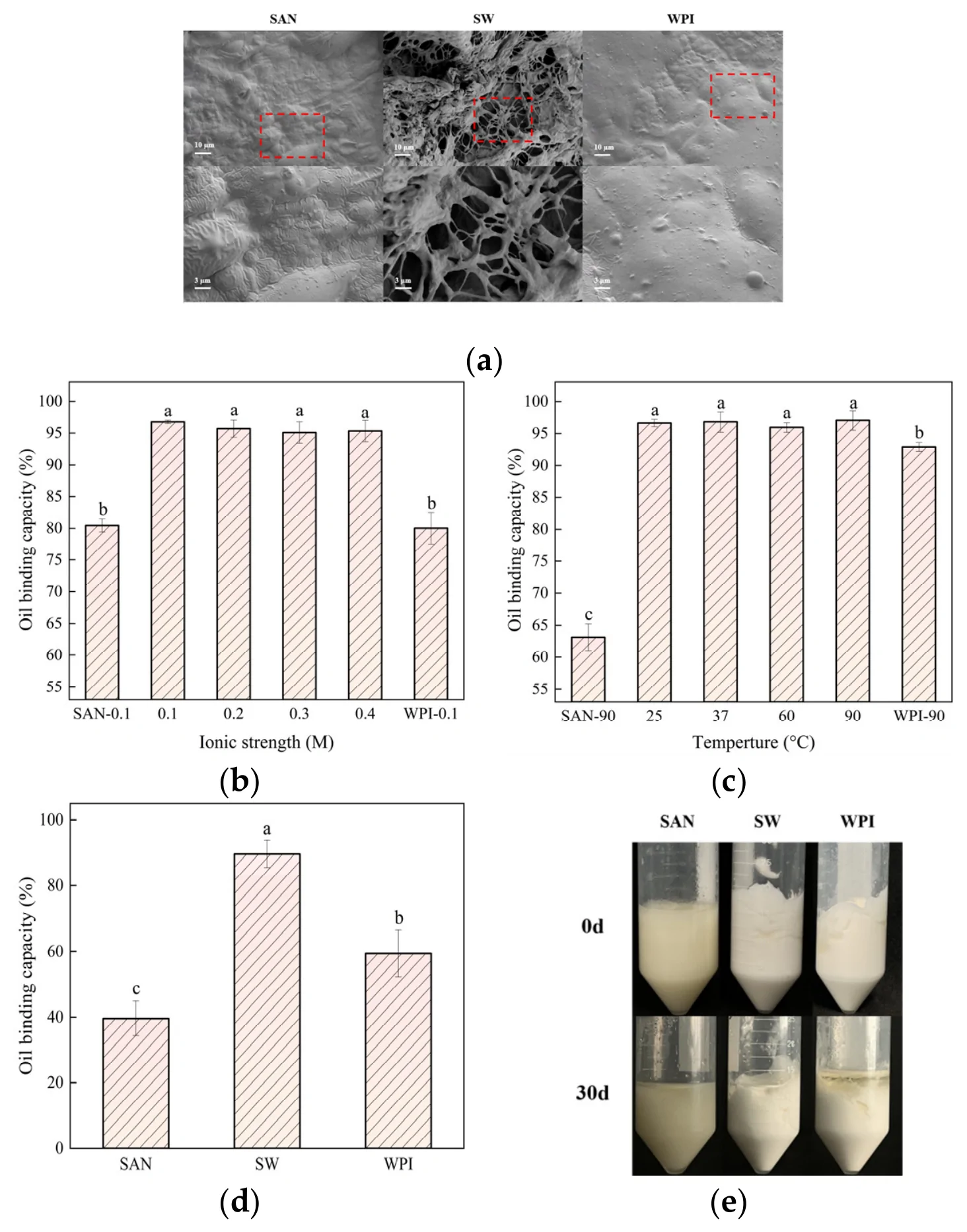
SW-HIPEs stability: SEM images of SW-HIPEs at 1k× magnification (top), with the red boxed area corresponding to the higher magnification (3k×) image shown below (bottom) (**a**). The OBC of ionic strength (0.1 M, 0.2 M, 0.3 M, and 0.4 M) (**b**). Thermal treatment (at 90 °C for 30 min) (**c**). Storage stability (at 4 °C for 30 days) (**d**). Storage visual appearance (**e**). Different small superscript letters (a–c) indicate the presence of statistically significant differences (*p* < 0.05).

Figure 3a presents the microstructure of the SW composite. Morphologically, SAN exhibits a loose, flaky structure with a rough surface, whereas the WPI consists of spherical protein particles with a relatively smooth surface. When WPI is blended with SAN, the WPI particles become embedded within the SAN structure, forming a continuous, uniform three-dimensional network characterized by a porous, honeycomb-like morphology. No significant structural collapse or phase separation is observed. This indicates that WPI and SAN interact after homogenization, which further contributes to the stabilization of HIPEs and provides a structural foundation for subsequent loading of additional oil droplets. These findings are consistent with those reported for the RP-pectin composite gels [37].

### 3.3. SW-HIPEs Stability

#### 3.3.1. Ionic Strength

Figure 3b illustrates the effect of ionic strength on the stability of the samples. Under high-salt conditions, the OBC of SW-HIPEs remains approximately 95%, with no significant change as the NaCl concentration increases. At 0.1 M NaCl, the OBC reaches 96.74%. Consistent with the findings of [38], both the trisaccharide gel and whey protein in this system carry a negative charge, resulting in natural electrostatic repulsion between molecules. Nevertheless, the emulsion remains highly stable; the entanglement of their molecular chains prevents the aggregation and coalescence of droplets, thereby maintaining the structural integrity of the HIPEs.

#### 3.3.2. Thermal Treatment

The OBC of SW-HIPEs at different temperatures is shown in Figure 3c. For WPI-HIPEs, heating induced protein aggregation, which unexpectedly increased the oil binding capacity to 92.88%. In contrast, SAN-HIPEs formed a heat-induced solid gel but remained unstable, with the oil binding capacity dropping sharply to 63.13%. Meanwhile, SW-HIPEs remained stable and maintained a gel-like state, and the OBC remained at 97.03%, with results consistent with those reported by [39]. This structure not only preserves the hydrophobic adsorption capacity of proteins toward oil droplets but also locks the oil phase through synergistic entrapment, ultimately maintaining a gel state even after compression. These results demonstrate that SW-HIPEs possess excellent heat resistance.

#### 3.3.3. Storage Stability

As shown in Figure 3d, after 30 days of storage at 4 °C, the OBC of HIPEs stabilized with SAN or WPI alone decreased sharply over the same period, accompanied by noticeable oil/water separation (Figure 3e), indicating that the structural integrity of the individual components had been compromised [40]. Meanwhile, the OBC of SW-HIPEs remained at 89.57%, which was 49.97% and 30.16% higher than those of single SAN and WPI, respectively. The above microstructural observation of the gel further confirmed that the combination of SAN and WPI synergistically improved the storage stability of HIPEs.

### 3.4. Encapsulation Efficiency (EE) and the OBC of SW-HIPEs-Cur

**Figure 4:**
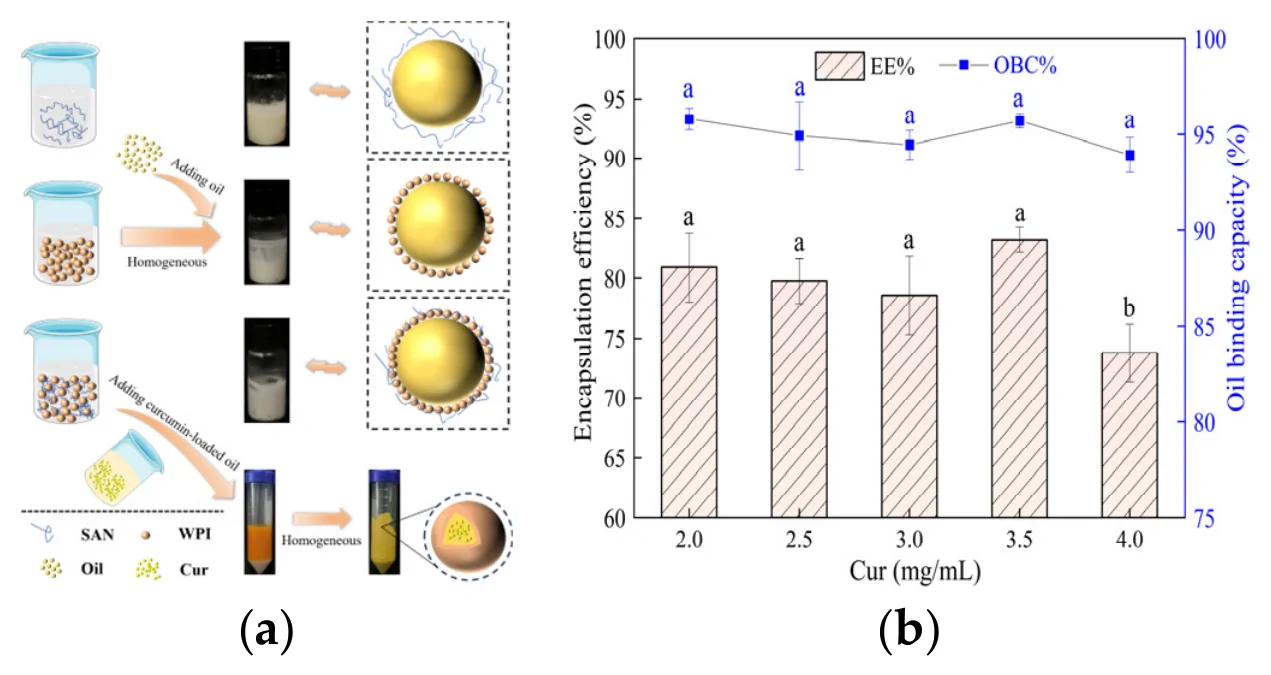
Schematic diagram of the preparation of SW-HIPEs-Cur (**a**) and the effect of different Cur concentrations on the EE and OBC of SW-HIPEs-Cur (**b**). Different small superscript letters (a,b) indicate the presence of statistically significant differences (*p* < 0.05).

The schematic diagram for the loading process of Cur into SW-HIPEs and the effects of different Cur concentrations on EE and OBC values of SW-HIPEs-Cur are displayed in Figure 4a,b. Within a Cur concentration range of 2–3.5 mg/mL, EE exceeding 80% is achieved, while the OBC remains stable at over 95%. The maximum EE of 83.23% is reached at a Cur concentration of 3.5 mg/mL. When the Cur concentration is further increased to 4%, the excess Cur occupies the polysaccharide–protein interface, causing the EE to drop significantly to 73.76%, whereas the OBC remains unchanged, indicating that the physical entrapment of oil droplets is still intact. In contrast, under the same loading conditions, the EE and OBC of the single WPI decreased to 68.73% and 83.58%, respectively. To balance high EE and high OBC, a Cur concentration of 3.5 mg/mL was selected for subsequent experiments.

### 3.5. FTIR

**Figure 5:**
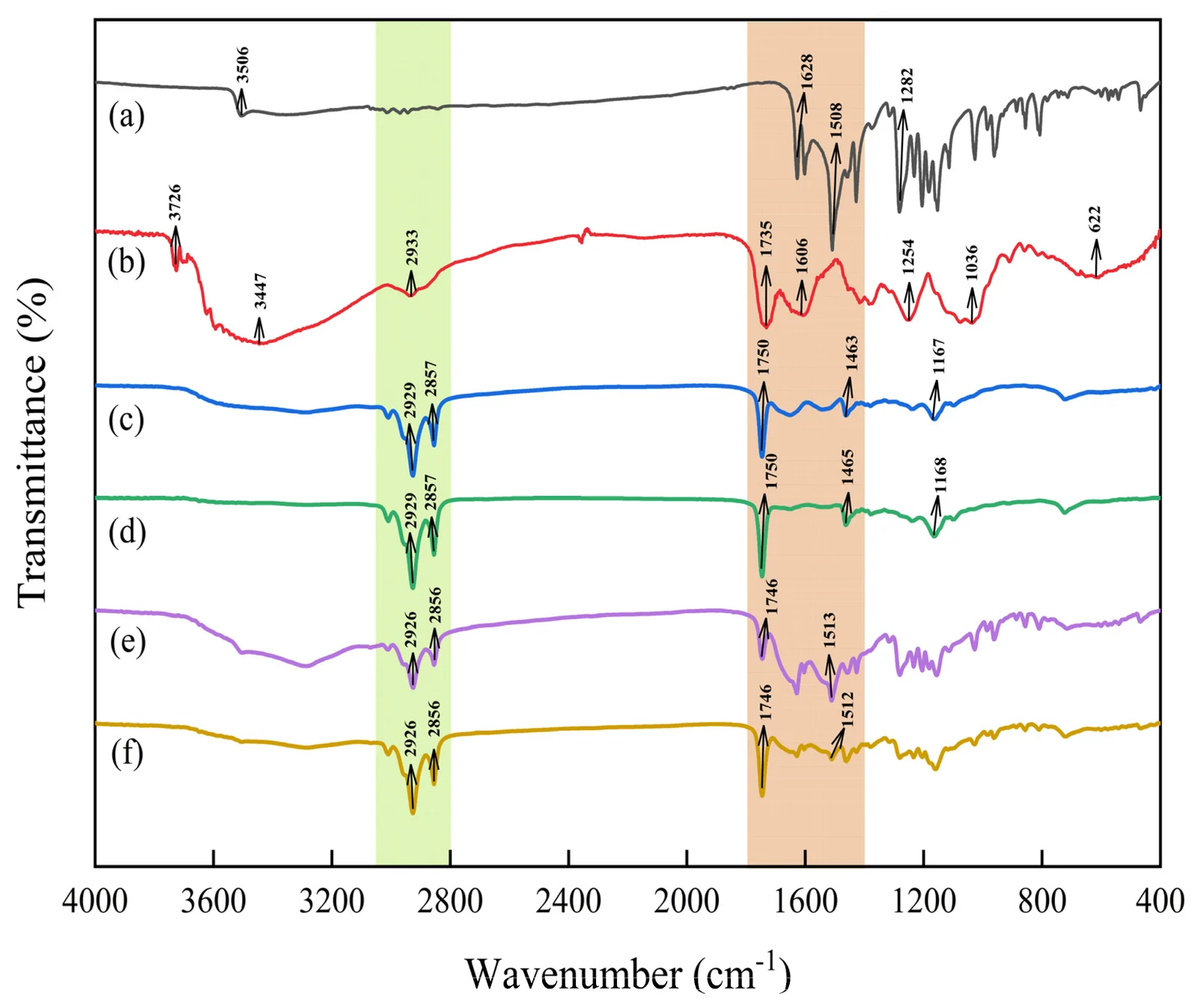
The FTIR spectra: Cur (**a**); SAN−HIPEs (**b**); WPI−HIPEs (**c**); SW−HIPEs (**d**); WPI−HIPEs−Cur (**e**); SW−HIPEs−Cur (**f**).

To further investigate the interactions among SAN, WPI, and the loaded Cur, the FTIR spectra of the samples are presented in Figure 5. Two characteristic absorption bands were observed: the amide A band (3500–3100 cm^−1^) and the amide I and II bands (1700–1400 cm^−1^) [41]. Upon complexation, the FTIR spectra of SW and WPI were similar, indicating that SAN and WPI interact primarily through non-covalent bonds. The amide A band corresponds to the stretching vibrations of O-H and N-H bonds [42]. Changes in its absorption peak suggest the formation of hydrogen bonds between the amino groups of WPI and the hydroxyl and carboxyl groups of SAN [43], with a decrease in band intensity indicating weakened hydrogen bonding. Consistent with the hydrophobicity data presented earlier, the addition of SAN increases the hydrophobicity of the complex. The pronounced vibrational changes in the 1750–1400 cm^−1^ region may be associated with conformational changes in the polypeptide chains [44]. In SW-HIPEs, the amide II band shifts from 1463 cm^−1^ to 1465 cm^−1^, indicating an alteration in protein secondary structure [45]. The non-covalent interactions in SW primarily include hydrophobic interactions, hydrogen bonds, and electrostatic interactions. The electrostatic force likely arises from interactions between the anionic -COO^−^ groups of SAN and the cationic -NH^+^ groups of WPI, which is similar to the findings of [46] regarding the interaction between SAN and Ca^2+^.

Cur displays a characteristic peak at 3506 cm^−1^, alongside several sharp peaks at 1628, 1508, and 1282 cm^−1^ assigned to vibrations of distinct functional groups [47]. Nearly all characteristic peaks of Cur vanished in the spectra of SW-HIPEs, which verified the successful loading of Cur molecules into the emulsion systems. The restriction of the stretching and bending vibrations of Cur molecules reflects the change in the molecular environment before and after encapsulation, as well as the interaction between Cur and the system [48]. These results are consistent with the findings of [49] on the effect of SAN on hemp protein plant-based cheese and those of [50] in Cur-loaded soy protein–polysaccharide composite emulsions.

### 3.6. SW-HIPEs-Cur Stability

#### 3.6.1. UV Light and Thermal Stability

**Figure 6:**
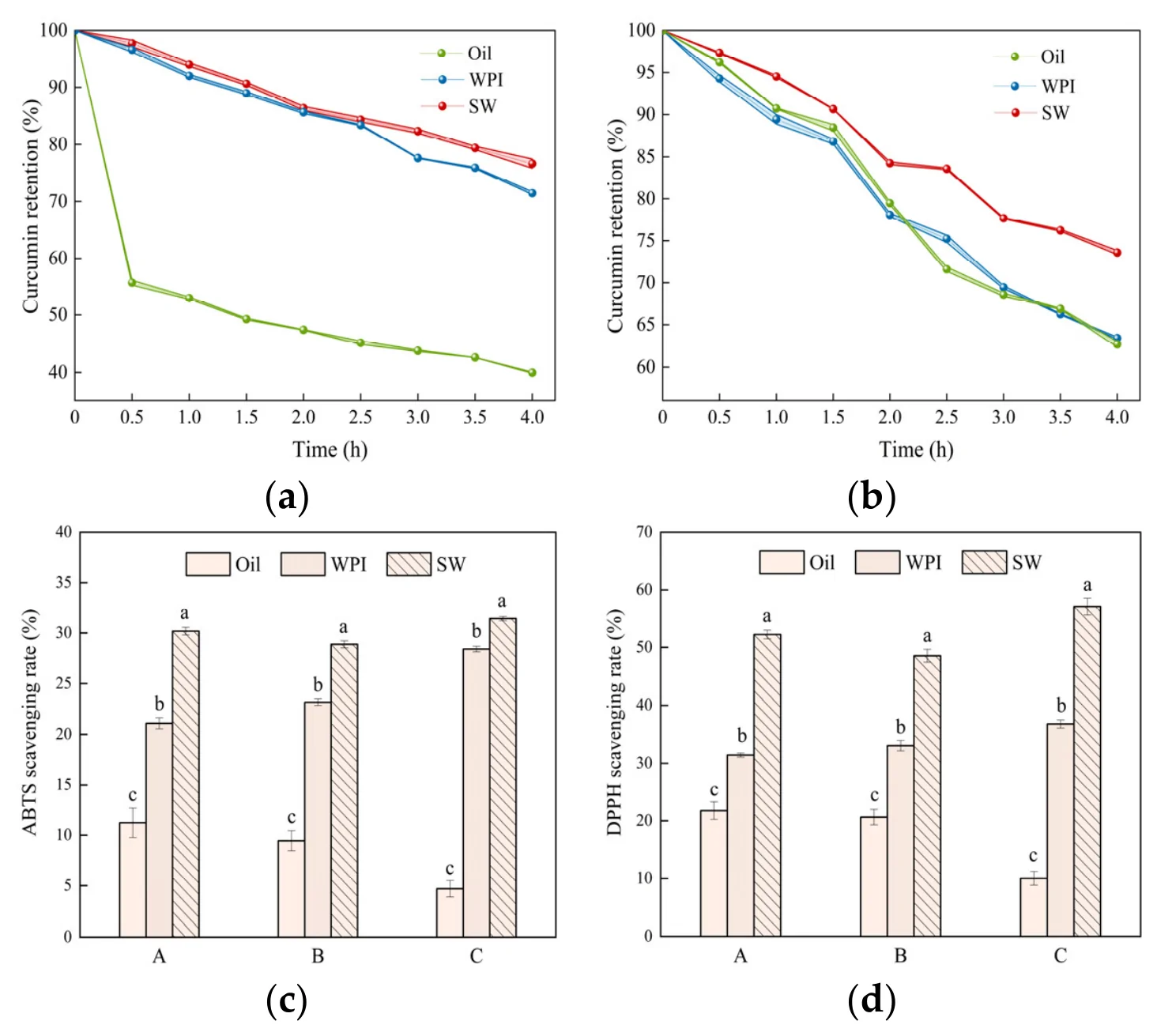
Cur retention rate after different stress treatments and antioxidant capacity of SW-HIPEs-Cur: UV irradiation (**a**). Heat-stressed (**b**). ABTS (**c**) and DPPH (**d**) scavenging rates. Here, A, B, and C represent the unstressed, salt-stressed (0.1 M), and heat-stressed (90 °C for 30 min) treatments. Different small superscript letters (a–c) indicate the presence of statistically significant differences (*p* < 0.05).

As a natural polyphenolic active substance, Cur exhibits poor environmental stability, and its biological activity is prone to degradation under conditions such as UV light and heating. As illustrated in Figure 6a, after 4 h of UV irradiation, the Cur retention rate of all samples decreased with prolonged irradiation time; the final Cur retention rates of oil, WPI-HIPEs, and SW-HIPEs were 39.96%, 71.47%, and 76.66%, respectively. The Oil–Cur phase, which was directly exposed to UV radiation without the protection of an interfacial barrier, exhibited the most rapid degradation. In contrast, WPI compounded with SAN exhibited better light stability, with Cur protected by the physical barrier formed by the composite shell. Cur had the slowest degradation rate due to physical barriers preventing its contact with the external environment [51], and its retention rate was significantly higher than that of Oil–Cur.

Figure 6b illustrates the changes in curcumin retention in HIPEs after heat treatment at 90 °C for 4 h. The results indicate that the Cur retention rate of SW-HIPEs decreased slowly, providing an effective guarantee for Cur delivery, with a final retention rate of 73.61%, which was significantly higher than that of oil (62.68%) and WPI-HIPEs (63.41%). Consistent with the findings of [52], the shell formed by SW composite particles coats the surface of Cur-loaded oil droplets, effectively shielding Cur from direct contact with external oxygen through an interfacial barrier, thereby reducing the oxidation rate.

#### 3.6.2. Antioxidant Capacities

The ABTS (Figure 6c) and DPPH (Figure 6d) radical scavenging rates of the oil, WPI, and SW systems loaded with Cur followed a similar trend. Compared with Oil–Cur, the WPI system enhanced the ABTS and DPPH scavenging rates by approximately 9.9% and 5.7%, respectively, while the SW system exhibited even greater increases, with ABTS and DPPH scavenging rates enhanced by approximately 19.0% and 26.5%, respectively. These improvements are primarily attributed to the enhanced dispersion and apparent solubility of Cur within HIPEs, which promote its accessibility to free radicals [53]. Additionally, the covalent interactions between WPI and Cur help form a compact aggregated structure, delaying Cur degradation [54]. Notably, compared with WPI-HIPEs-Cur, the SW-HIPEs-Cur further increased the ABTS scavenging rate by approximately 10% and showed an even more pronounced improvement in DPPH scavenging, indicating that the combination of SAN and WPI not only optimizes the emulsion interface and improves physical stability but also contributes to the enhanced antioxidant performance of Cur, likely by protecting Cur from degradation through the formation of a dense interfacial network. Under environmental stress from ionic strength and heating, Oil–Cur undergoes significant structural degradation and a substantial reduction in antioxidant activity. In contrast, HIPEs possess a dense three-dimensional network with strong resistance to salt ion interference; moreover, heat treatment induces thermal denaturation of WPI, which, together with the thermal gelation properties of SAN, further enhances the compactness of the emulsion gel network. Consequently, Cur-loaded emulsions retain high antioxidant activity even after heat stress treatment and, in some cases, exhibit increased activity [55].

### 3.7. Stability and Bioaccessibility of SW-HIPEs-Cur In Vitro Digestion

**Figure 7:**
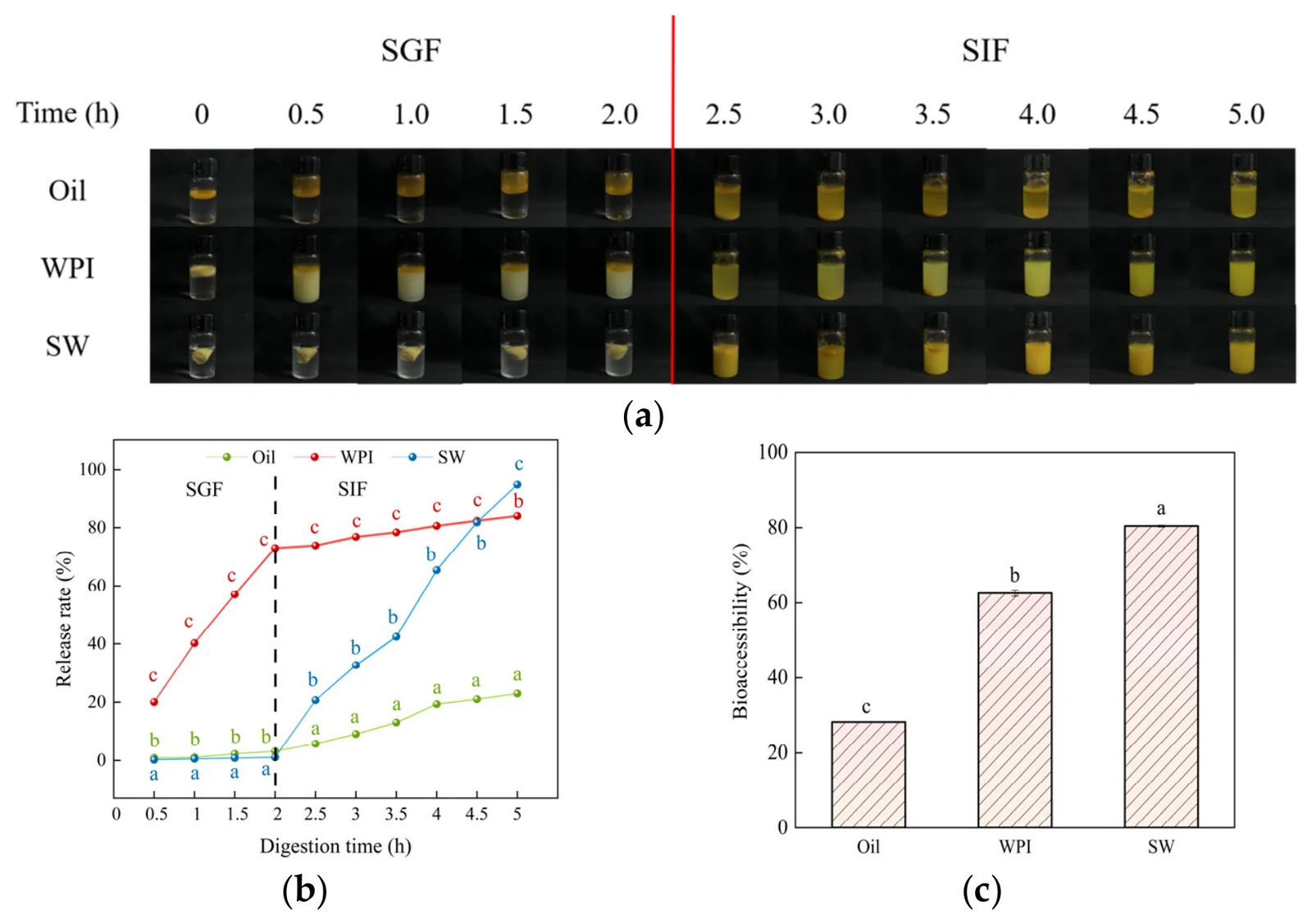
Release characteristics of SW-HIPEs-Cur: Morphological evolution during gastrointestinal digestion (**a**). Cur release rate (**b**). Bioaccessibility (**c**). Different small superscript letters (a–c) indicate the presence of statistically significant differences (*p* < 0.05).

In vitro simulated digestion is an important method for studying drug release. Due to the instability of Cur in the gastrointestinal tract, its bioactivity is reduced, resulting in low bioaccessibility. Therefore, this study investigated the effect of SW-HIPEs on the Cur release rate through in vitro gastrointestinal digestion experiments. Figure 7a shows the dynamic behavior of Cur in gastric and intestinal environments. During gastric digestion, the Oil–Cur was observed to float on top of the simulated gastric fluid (SGF), with only a small amount of Cur released. In the WPI-HIPEs, as digestion time increased, the gel disintegrated, leading to substantial Cur release that became uniformly dispersed throughout the SGF. Owing to the acid stability of SAN, SW-HIPEs retained their morphological integrity over prolonged digestion and remained suspended in the upper layer of SGF with a low Cur release rate of 1.06%, which was 71.91% lower than that of the WPI system and 2.05% lower than that of the Oil–Cur in gastric digestion. During intestinal digestion, benefiting from the pH responsiveness of SAN, SW-HIPEs exhibited a sustained and slow release behavior, with a final release rate reaching 94.77%, which was 10.74% higher than the WPI system and 71.83% higher than the Oil–Cur system. Such unique release behavior distinguishes SW-HIPEs from conventional single protein or polysaccharide delivery systems.

Figure 7b shows the in vitro release profiles of Cur from HIPEs in simulated gastrointestinal fluids. In the WPI, substantial Cur release in the gastric fluid led to an early plateau in release upon entering the intestinal fluid. In contrast, Cur in the SW-HIPEs was released slowly during the intestinal digestion stage. Similar to previous findings in [28], the addition of SAN reinforced the network structure of the HIPEs, making it more compact and effectively hindering the diffusion of bile salts and digestive enzymes into the gel, reducing the gel dissolution rate and impeding Cur release. As summarized in Table 1 from other reports, when WPI is used as a single stabilizer, a concentration of 10 wt% is required to achieve stable HIPEs with 90% oil phase. In contrast, SW-HIPEs, with only 3% WPI and 80% oil phase, achieved a higher loading concentration of Cur while maintaining good encapsulation efficiency compared with other systems. Figure 7c illustrates that the final bioaccessibility of Cur in SW-HIPEs was 80.46%, which was markedly higher than those determined for WPI-HIPEs (62.61%) and oil (28.27%). Overall, these results indicate that the gel network structure delays Cur degradation during in vitro digestion, allowing slow release of Cur into the simulated fluid as lipase contacts the oil droplet surface. Furthermore, the interaction mechanism between SAN, WPI, and Cur molecules requires further investigation.

## 4. Conclusions

In this study, low-protein HIPEs (SW-HIPEs) stabilized by SAN and WPI were constructed, with their environmental stability, curcumin encapsulation efficiency, protective capability, and in vitro gastrointestinal release rate comprehensively explored. Benefiting from the unique physicochemical properties of SAN, the synergistic interaction between SAN and WPI formed a stable and highly viscoelastic interfacial network, which endowed SW-HIPEs with excellent environmental tolerance. The results showed that the optimized composite HIPE, constructed with 0.8% SAN and a low WPI dosage of 3%, could efficiently load Cur at 3.5 mg/mL, achieving a high encapsulation efficiency of 83.23%. The fabricated SW-HIPEs effectively suppressed Cur degradation under UV irradiation and high-temperature conditions, improving Cur retention by 1.8-fold and 2.1-fold, respectively. Meanwhile, the composite system increased the ABTS and DPPH radical scavenging rates of Cur by approximately 36.94% and 66.22%, and maintained superior antioxidant performance even under thermal and saline stress. In vitro digestion tests confirmed that the SW-HIPE network remained intact in the gastric phase, with a minimal Cur release rate of 1.06%. Cur was gradually and sustainably released in intestinal fluid, achieving a cumulative release rate of 94.77% and a final bioaccessibility of 80.46%. Overall, SW-HIPEs integrate low-protein formulation, high loading capacity, and excellent stability, enabling efficient encapsulation, protection, and controlled release of Cur. However, challenges remain for practical application, including stabilization mechanisms under complex conditions, compatibility with food matrices, and long-term protective effects on lipophilic bioactives. Future studies will address these issues to advance clean-label emulsion delivery systems in functional foods. In parallel, further validation in animal models is warranted to better understand the in vivo absorption and transport behavior of curcumin delivered by this system.

## Figures and Tables

**Table 1:** Results of Cur loading in emulsions with different stabilizers.

Stabilizer	Protein Content (*w*/*v*)	Oil Category (%)	Load Capacity (mg/mL)	EE (%)	Stomach (%)	Intestine (%)	Bioaccessibility (%)	Ref.
Pectin/ovalbumin	1	Refined olive oil (75)	1	\	\	70.8	38.8	[56]
Lycium barbarum polysaccharide/Wheat gluten amyloid fibrils	4	Soybean oil (80)	2	\	\	\	61.38	[57]
κ-carrageenan/pea protein isolate	5	Soybean oil (75)	1	83.54	\	\	\	[58]
Oligochitosan/whey protein isolate	8	Soybean oil (17)	\	96.64	\	41.74	40.34	[51]
Whey protein isolate	10	Fish oil (90)	1	\	\	65.6	77.4	[59]
Sanxan/whey protein isolate	3	Soybean oil (80)	3.5	83.23	1.06	94.77	80.46	This work

## Data Availability

The original contributions presented in this study are included in the article. Further inquiries can be directed to the corresponding authors.

## Abbreviations

The following abbreviations are used in this manuscript:

HIPEs	High internal phase emulsions
SAN	Sanxan
Cur	Curcumin
WPI	Whey protein isolate
SW	SAN-WPI
